# Paradigms, innovations, and biological applications of RNA velocity: a comprehensive review

**DOI:** 10.1093/bib/bbaf339

**Published:** 2025-07-16

**Authors:** Yajunzi Wang, Jing Li, Haoruo Zha, Shuhe Liu, Daiyun Huang, Lei Fu, Xin Liu

**Affiliations:** Wisdom Lake Academy of Pharmacy, Xi'an Jiaotong-Liverpool University, 111 Ren'ai Road, Suzhou Industrial Park, Suzhou, Jiangsu 215123, China; Institute of Systems, Molecular and Integrative Biology, University of Liverpool, Biosciences Building, Crown Street, Liverpool L69 7ZX, United Kingdom; Wisdom Lake Academy of Pharmacy, Xi'an Jiaotong-Liverpool University, 111 Ren'ai Road, Suzhou Industrial Park, Suzhou, Jiangsu 215123, China; Institute of Systems, Molecular and Integrative Biology, University of Liverpool, Biosciences Building, Crown Street, Liverpool L69 7ZX, United Kingdom; State Key Laboratory of Common Mechanism Research for Major Diseases, Suzhou Institute of Systems Medicine, Chinese Academy of Medical Sciences & Peking Union Medical College, 100 Chongwen Road, Suzhou Industrial Park, Suzhou, Jiangsu 215123, China; Wisdom Lake Academy of Pharmacy, Xi'an Jiaotong-Liverpool University, 111 Ren'ai Road, Suzhou Industrial Park, Suzhou, Jiangsu 215123, China; Institute of Systems, Molecular and Integrative Biology, University of Liverpool, Biosciences Building, Crown Street, Liverpool L69 7ZX, United Kingdom; Institute of Systems, Molecular and Integrative Biology, University of Liverpool, Biosciences Building, Crown Street, Liverpool L69 7ZX, United Kingdom; Department of Biological Sciences, School of Science, Xi'an Jiaotong-Liverpool University, 111 Ren'ai Road, Suzhou Industrial Park, Suzhou, Jiangsu 215123, China; Suzhou Municipal Key Lab of AI4Health, 111 Ren'ai Road, Suzhou Industrial Park, Suzhou, Jiangsu 215123, China; Wisdom Lake Academy of Pharmacy, Xi'an Jiaotong-Liverpool University, 111 Ren'ai Road, Suzhou Industrial Park, Suzhou, Jiangsu 215123, China; Wisdom Lake Academy of Pharmacy, Xi'an Jiaotong-Liverpool University, 111 Ren'ai Road, Suzhou Industrial Park, Suzhou, Jiangsu 215123, China; Wisdom Lake Academy of Pharmacy, Xi'an Jiaotong-Liverpool University, 111 Ren'ai Road, Suzhou Industrial Park, Suzhou, Jiangsu 215123, China

**Keywords:** RNA velocity, single-cell RNA sequencing, dynamic transcriptomics, computational modeling, advanced inference strategies

## Abstract

Single-cell RNA sequencing enables unprecedented insights into cellular heterogeneity and lineage dynamics. RNA velocity, by modeling the temporal relationship between spliced and unspliced transcripts, extends this capability to predict future transcriptional states and uncover the directionality of cellular transitions. Since the introduction of foundational frameworks such as *Velocyto* and *scVelo*, an expanding array of computational tools has emerged, each based on distinct biophysical assumptions and modeling paradigms. To provide a structured overview of this rapidly evolving field, we categorize RNA velocity models into three classes: steady-state methods, trajectory methods, and state extrapolation methods, according to their underlying approaches to transcriptional kinetics inference. For each category, we systematically analyze both the overarching principles and the individual methods, comparing their assumptions, kinetic models, and computational strategies and assessing their respective strengths and limitations. To demonstrate the biological utility of these tools, we summarize representative applications of RNA velocity across developmental biology and diseased microenvironments. We further introduce emerging extensions of RNA velocity methods that go beyond classical splicing kinetics. Finally, we discuss existing limitations regarding model assumptions, preprocessing procedures, and velocity visualization and offer practical recommendations for model selection and application. This review offers a comprehensive guide to the RNA velocity landscape, supporting its effective implementation in dynamic transcriptomic research.

## Background

Single-cell RNA sequencing (scRNA-seq) has revolutionized the study of biological systems by enabling the exploration of cellular heterogeneity, lineage tracing, and gene regulatory network dynamics at an unprecedented resolution [1–3]. This technology has provided critical insights into complex biological processes such as cellular development, differentiation, immune response, and tumor evolution, thereby paving the way for transformative advances in the field of developmental biology [3, 4]. The analysis of cellular development and differentiation presents a unique challenge, as cells traverse a continuous landscape of states rather than existing in discrete categories. Traditional trajectory inference methods reconstruct developmental paths by ordering cells according to their transcriptional similarity. This approach effectively creates a pseudotemporal sequence that reflects biological progression [3, 5, 6]. However, these methods are limited by the static nature of single-cell measurements, which only capture snapshots of cellular states. While trajectory inference has proven valuable for understanding cell fate decisions, the selection of appropriate methods depends heavily on the expected trajectory topology (linear, branching, or cyclic) and selected methods often require validation through multiple approaches [7].

RNA velocity analysis represents a significant advancement in this field, offering a more direct way to infer cellular dynamics. Unlike traditional trajectory inference methods that rely solely on transcriptional similarities, RNA velocity leverages the relative abundance of spliced and unspliced messenger RNA (mRNA) to predict future cell states. This approach, first introduced by La Manno *et al*. [8], captures directed dynamic information and predicts future cell states by distinguishing the relative abundances of unspliced pre-mRNA and spliced mature mRNA presented in single-cell RNA sequencing (scRNA-seq), based on a steady-state theory. By modeling transcriptional dynamics with a system of ordinary differential equations (ODEs), RNA velocity infers the instantaneous rate of change in unspliced mRNA abundance (*ds*/*dt*) for individual genes from relative mRNA abundances, known as RNA velocity. A positive RNA velocity indicates an induction in transcriptional state, and a negative velocity indicates gene repression. This qualitative premise has profound implications for the analysis of scRNA-seq data. The experimentally observed transcriptome is a snapshot of a biological process [9]. By integrating snapshot data with a causal model, it has become possible to reconstruct both the dynamics and direction of this process without prior knowledge or specialized experiments.

The first RNA velocity model, *Velocyto*, developed by La Manno *et al*. [8], captures directed dynamic information and predicts future cell states. It works by distinguishing the relative abundances of unspliced pre-mRNA and spliced mature mRNA detected in scRNA-seq, based on steady-state theory. This foundational framework was subsequently enhanced by Bergen *et al*. [10] in *scVelo*, which implemented a more sophisticated dynamical model capable of inferring transcriptional dynamics and assigning latent cell time. Together, *Velocyto* and *scVelo* have become cornerstone tools in RNA velocity analysis. Several reviews have provided comprehensive comparisons of them, delving into implementation details and discussing valuable aspects of data processing and visualization techniques [11–13]. Followed by *Velocyto* and *scVelo*, numerous advanced models [8, 10, 14–26] have been developed, each leveraging distinct computational frameworks grounded in specific biophysical assumptions.

Despite the rapid advancement of RNA velocity models, no comprehensive review has yet provided a thorough comparison of their strengths, limitations, and applicability. This review aims to address this gap by providing a structured and in-depth analysis of existing RNA velocity computational tools. We categorize these tools into three main classes based on their approaches to learning transcriptional kinetics. For each category, we compare and contrast the underlying assumptions, kinetic formulations, and computational strategies, highlighting the unique strengths and limitations of each method. To illustrate their biological utility, we summarize key applications of RNA velocity. Furthermore, we discuss emerging extensions of RNA velocity methods that expand beyond traditional splicing kinetics. Finally, we address the current challenges in RNA velocity analysis, such as model assumptions, preprocessing pipelines, and velocity visualization, and offer practical recommendations for selecting and applying these tools effectively in both basic and translational research.

## Workflow and implementation

**Figure 1:**
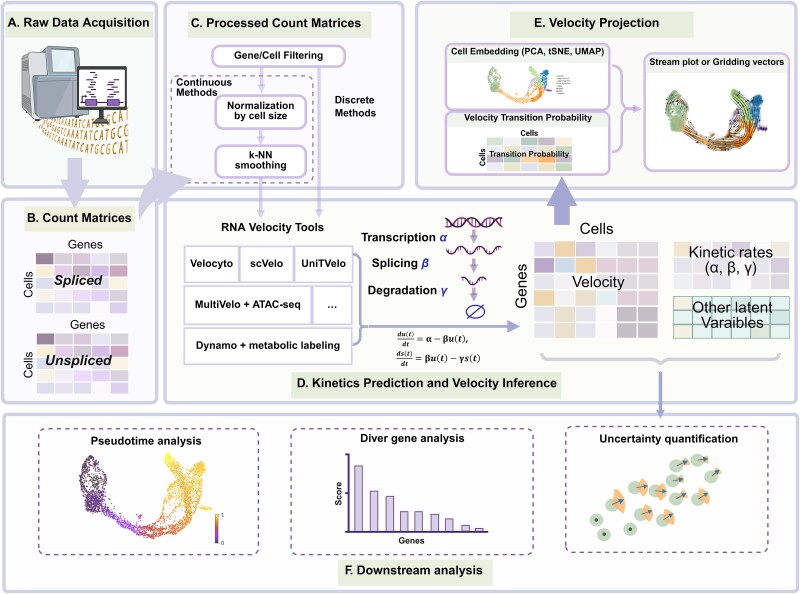
Workflow for RNA velocity analysis. (A) Raw scRNA-seq data acquisition. (B) Quantification of unspliced and spliced transcript abundances. (C) Count matrices preprocessing, data normalization, and neighborhood smoothing are included in the classic workflow. (D) Estimation of RNA velocities by fitting spliced and unspliced counts to biophysical models, also yielding kinetic parameters and latent variables. (E) Visualization of high-dimensional velocity vectors in low-dimensional space via methods such as streamline plots and grid-averaged vector fields. (F) Downstream analyses.

The typical RNA velocity analysis pipeline (Fig. 1A) begins with an essential preprocessing step to distinguish between unspliced and spliced transcripts in the raw sequencing data. Several tools [8, 27–32] have been developed to quantify these abundances (Fig. 1B), enabling the construction of separate count matrices [33]. Given the inherent noise in single-cell RNA sequencing data, most analytical frameworks employ sophisticated data smoothing techniques (also known as data imputation) to extract reliable signals for velocity inference (Fig. 1C). A prevalent approach involves computing the first-order moment (mean) across k-nearest neighbors (KNN) in the expression space. While the majority of protocols incorporate additional preprocessing steps, including library size normalization and log-transformation [11], the choice between using such processed data versus raw count data can influence RNA velocity results and is often dependent on the specific inference model employed. For instance, some alternative estimation frameworks (e.g. *TopicVelo* [16], *Pyro-Velocity* [22], and *cell2fate* [23]) are specifically designed to treat transcript dynamics as a discrete stochastic process, allowing the direct use of raw count matrices. This approach may preserve biological signals, including informative noise and gene–gene interactions, while minimizing potential parameter estimation biases that might arise from extensive preprocessing (further explored in the Discussion section Navigating Challenges and Toward Best Practices) [11]. The subsequent phase involves estimating cell-specific RNA velocities in high-dimensional space by applying various biophysical models to fit unspliced and spliced transcript counts (Fig. 1D). This process yields essential kinetic parameters, including transcription, splicing, and degradation rates. Depending on the chosen modeling framework, additional latent variables such as latent time can be simultaneously inferred, further enhancing the resolution of transcriptional dynamics.

Once high-dimensional velocity vectors are derived, they are used to calculate transition probabilities and then projected into a low-dimensional representation using methods such as Uniform Manifold Approximation and Projection (UMAP) [34], t-distributed Stochastic Neighbor Embedding (t-SNE) [35], and Principal Component Analysis (PCA) [36] (Fig. 1E). The projected velocities are commonly visualized through techniques like streamline plots or grid-averaged vector fields.

This workflow was originally proposed by La Manno *et al*. [8] and subsequently refined by Bergen *et al*. in the *scVelo*’s framework [10]. Most later RNA velocity tools adopted this general paradigm, differing primarily in their approaches to kinetic prediction and velocity inference. Recent advances have further redefined the field by incorporating biophysically grounded dynamic processes and optimizing computational frameworks for learning and interpreting transcriptional dynamics. These conceptual and technical advances have enabled RNA velocity frameworks to support increasingly sophisticated downstream analyses, extending beyond velocity estimation to facilitate the interpretation of cellular dynamics and the underlying molecular mechanisms (Fig. 1F). Many RNA velocity models integrate inferred kinetic parameters and latent variables to support downstream analyses that provide deeper insights into dynamic processes and potential regulatory mechanisms. During the estimation phase, several models infer latent time alongside kinetic parameters [10, 14, 17, 19–23]. These temporal measurements serve as a cellular internal clock, precisely tracking cell progression through biological processes [6, 7]. Various analytical tools also offer specialized functionalities. For instance, *scVelo* can identify key driver genes orchestrating the transcriptional dynamics captured in velocity estimates. Bayesian models provide additional insights by quantifying posterior uncertainty in velocity and kinetic parameter estimates, thereby enabling robust statistical assessment [19–23, 25]. In addition, the RNA velocity framework has led to the development of numerous specialized computational tools for trajectory analysis [37–41] and enhanced visualization [42–44], alongside benchmarking tools [45, 46] that generate synthetic scRNA-seq data mimicking cellular developmental trajectories. These advancements support rigorous model validation and dynamic process reconstruction, reinforcing RNA velocity as a powerful tool in single-cell analysis.

In the following sessions, we review existing representative RNA velocity methods, providing an extensive overview of current developments in RNA velocity computational models. We categorize these methods according to their paradigms for modeling transcriptional dynamics and systematically dissect their kinetic prediction and velocity inference strategies, along with the underlying biophysical assumptions. Furthermore, we map their applications to critical biological scenarios and discuss the current challenges these methods are facing.

## Kinetics prediction and velocity inference

**Figure 2:**
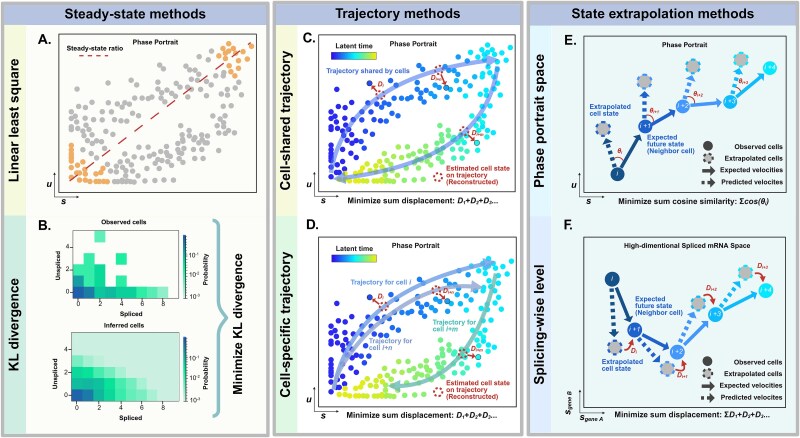
RNA velocity methods are categorized into three classes based on their paradigms in learning transcriptional dynamics. (A, B) Steady-state methods, include linear regression based on the steady-state ratio and inference based on minimizing Kullback–Leibler (KL) divergence between observed and predicted distributions. (C, D) Trajectory-based methods, where either cell-shared or cell-specific latent trajectories are used to reconstruct cellular dynamics by minimizing the sum of displacements between observed and estimated states. (E, F) State extrapolation methods, which infer future states by minimizing cosine similarity or distance in phase portrait space or high-dimensional gene space.

Based on distinct paradigms in learning transcriptional dynamics, we categorize RNA velocity methods into three classes: steady-state methods, trajectory methods, and state extrapolation methods (Fig. 2 and Table 1). Steady-state methods, such as *Velocyto*, solve transcriptional dynamics relying on a steady-state assumption. These methods typically presume a constant splicing rate and infer kinetic rates using steady-state subpopulations. Trajectory methods, exemplified by *scVelo*, estimate kinetic parameters to construct phase portrait trajectories that align observed cells with their respective corresponding cell times. State extrapolation methods leverage expected future cell states to guide the estimation and optimization of cell-level RNA velocity vectors. In the following sections, we introduce and compare representative RNA velocity models, with a focus on their design of computational frameworks as well as underlying modeling concepts (Table 2).

**Table 1:** Summary of three categories RNA velocity computational methods.

**Category**	**Methods**	**Modeling approach**	**Advantages**	**Limitations**
Steady-state methods	*Velocyto*, *scVelo* (deterministic and stochastic model), *MultiVelo* (deterministic and stochastic model), *VeloAE*, *TopicVelo*	Analytical or stochastic models assuming a constant splicing rate and transcriptional equilibrium. Typically use least-squares regression on steady-state cells.	Simple, fast, and interpretable; Effective in capturing clear steady-state differentiation processes.	Assumes constant splicing rate; Assumptions often violated in heterogeneous populations or when dynamics are incomplete; Inaccurate for complex kinetic patterns and nonsteady states.
Trajectory methods	*scVelo* (dynamical model), *MultiVelo* (dynamical model), *UniTVelo*, *Dynamo*, *veloVI*, *VeloVAE*, *LatentVelo*, *Pyro-Velocity*, *cell2fate*	Fit full transcriptional dynamics by constructing phase trajectories using ODEs; often involve latent time and EM or VAE frameworks.	Flexible modeling of complex, nonlinear dynamics; Generate biologically meaningful latent variables during inference; Often include uncertainty estimates.	Sensitive to incomplete trajectories; Kinetics inference is restricted by ODE formulation; Complex optimization.
State extrapolation methods	*cellDancer*, *DeepVelo*, *SymVelo*	Local modeling of cell-specific kinetics via nearest neighbors; learn velocity by extrapolating expression states over time in high-dimensional space.	Superior ability to capture lineage heterogeneity and subtle kinetic variations; Provide cell-specific kinetic parameters.	Limited practical validation in biological settings; Sensitive to local neighboring cell identification; Computationally intensive.

**Table 2:** Summarization of RNA velocity models.

**Category**		**Models**	**Multi-omics**	**Discrete raw count use**	**Gene-specific kinetic parameter**	**Gene-specific transcription rate**	**Gene-specific splicing rate**	**Gene-specific degradation rate**	**Latent time in parameter estimation**	**Transcriptional dynamics modeling**	**Parameter estimation framework**	**Ref.**
**Steady-state methods**	**Linear regression**	*Velocyto* *(scVelo–Deterministic)*	No	No	Yes	Yes	Global constant	Yes	No	Linear ODE	Linear least squares	[8, 10]
		*scVelo (Stochastic)*	No	No	Yes	Yes	Global constant	Yes	No	Linear ODE with second-order moments	Linear least squares	[10]
		*MultiVelo (Stochastic)*	ATAC-seq	No	Yes	Yes	Global constant	Yes	No	Linear ODE with second-order moments	Linear least squares	[14]
		*VeloAE*	No	No	Yes (latent space)	Yes (latent space)	Global constant	Yes (latent space)	No	Linear ODE in latent space	Autoencoder	[15]
	**KL divergence**	*TopicVelo*	No	Yes	Process-specific	Process-specific	Global constant	Process-specific	No	Chemical master equation	Backpropagation	[16]
**Trajectory methods**	**Cell-shared trajectory**	*scVelo (Dynamical)*	No	No	Yes	Yes	Yes	Yes	Gene-specific	Linear ODE	Expectation maximization	[10]
		*MultiVelo (Dynamical)*	ATAC-seq	No	Cell-specific	Cell-specific	Yes	Yes	Gene-specific	Linear ODE with chromatin accessibility	Expectation maximization	[14]
		*UniTVelo*	No	No	Yes	No TR	Yes	Yes	Gene-specific or shared	RBF-based ODE	Expectation maximization	[17]
		*Dynamo*	Metabolic labeling	No	Yes	Yes	Yes	Yes	Gene-specific labeling time	Linear ODE	Nonlinear least squares	[18]
		*Pyro-Velocity*	No	Yes	Yes	Yes	Yes	Yes	Gene-shared	Linear ODE	Stochastic variational inference	[22]
	**Cell-specific trajectory**	*veloVI*	No	No	Cell-specific*	Cell-specific*	Yes	Yes	Gene-specific	Linear ODE with transcription regulation	Variational autoencoder	[19]
		*VeloVAE*	No	No	Cell- & lineage-specific	Cell-specific	Lineage-specific	Lineage-specific	Gene-shared	Linear ODE with transcription regulation	Variational autoencoder	[20]
		*LatentVelo*	ATAC-seq*	No	Cell-specific (latent space)	No TR	No SR	No DR	Gene-shared	Neuro ODE with transcription regulation in latent space	Variational autoencoder	[21]
		*cell2fate*	No	Yes	Cell-specific	Cell-specific	Cell-specific*	Cell-specific*	Gene-shared	Linear ODE with transcription regulation	Stochastic variational inference	[23]
**State extrapolation method**	**Cosine similarity**	*cellDancer*	No	No	Cell-specific	Cell-specific	Cell-specific	Cell-specific	No	Linear ODE	Backpropagation	[24]
	**State distance**	*DeepVelo*	No	No	Cell-specific	Cell-specific	Cell-specific	Cell-specific	No	Linear ODE	Backpropagation	[25]
		*SymVelo*	ATAC-seq*	No	Cell-specific	No TR	No SR	No DR	No	Neuro ODE	Mutual learning (state extrapolation module & steady-state module)	[26]
					Yes (latent space)	Yes (latent space)	Global constant	Yes (latent space)	No	Linear ODE in latent space		

Abbreviation: No TR, No SR, and No DR indicate transcription rate, splicing rate, and degradation rate are not explicitly modeled in the ODE system (e.g. hyper kinetic parameters in neuro ODE system). Descriptions with *stars* (*) refer to features in model extension.

### Steady-state methods

La Manno *et al*. [8] proposed the first RNA velocity estimation framework, *Velocyto*, which is grounded in a steady-state assumption. This assumption posits that after transcriptional initiation, the abundances of unspliced and spliced mRNA for genes reach a steady-state equilibrium due to ongoing molecular degradation. When gene expression ceases, mRNA abundance gradually decays to zero. *Velocyto* introduces strict constraints on kinetic parameters in the rate equation (Supplementary Material 1). It treats transcription and degradation rates as time-dependent variables to be estimated while defining the splicing rate as a fixed constant (typically 1) across all genes. This simplification helps to reduce model complexity and ensure tractability. For each gene, steady-state cells, where unspliced and spliced mRNA abundances reach minimal or maximal values, are identified (Fig. 2A). A least-squares linear regression is then applied to these steady-state cells to analytically derive the degradation rate, also referred to as the steady-state ratio. Subsequently, RNA velocity is computed for each cell using the derived kinetic parameters and a closed-form equation (Fig. 1D).

Inspired by *Velocyto*, Bergen *et al*. [10] developed *scVelo*, a comprehensive and extensible computational framework that integrates the original steady-state model proposed by *Velocyto* and extends it by incorporating a stochastic model. This stochastic model reformulates the first-order transcriptional ODEs by including second-order moments (variance and covariance of unspliced and spliced counts) to account for stochasticity. Similar to the steady-state approach, a least-squares fit is employed to estimate degradation rates from steady-state populations. Subsequently, *MultiVelo* [14] further advanced these models by integrating chromatin accessibility information derived from Assay for Transposase-Accessible Chromatin using sequencing (ATAC-seq) data [47]. In MultiVelo, transcriptional regulation is simplified by abstracting various regulatory elements (such as chromatin modifiers, pioneer factors, and transcription factors) into a single rate parameter reflecting chromatin accessibility. Such transcriptional regulation is explicitly modeled by linking transcription rates to chromatin accessibility within the ODE framework, thereby enabling a more accurate and biologically informed estimation of kinetic parameters.

*VeloAE* [15] solves the steady-state ratio and infers latent transcriptional dynamics within a learned low-dimensional representation using an autoencoder framework. Specifically, it uses a graph convolutional network (GCN) to smooth the pre-encoded latent cell states (latent unspliced and spliced mRNA). It then adopts an attentive combination module in the decoder to reconstruct the input mRNA abundances. The autoencoder framework allows *VeloAE* to capture biologically meaningful latent cell states by minimizing the reconstruction loss between input and output count matrices. By learning kinetics rates and RNA velocity in a low-dimensional space, *VeloAE* is able to recover denoised transcriptional dynamics and mitigates the sparsity challenges that are often encountered in high-dimensional raw count data.

In contrast to other steady-state models, *TopicVelo* [16] employs a probabilistic topic modeling framework to disentangle potentially simultaneous processes. This model assumes that multiple biological processes, also referred to as topics (e.g. proliferation, immune response, and system-specific processes), can be identified from scRNA-seq by adopting Bayesian Nonnegative Matrix Factorization (BNMF). This approach identifies both process-specific gene signatures and cell-specific topic activity levels. Operating directly on raw count data, *TopicVelo* incorporates transcriptional bursting into a chemical master equation (CME) framework (Supplementary Material 1). The Gillespie algorithm [48] is then applied to each gene within each process to simulate the stochastic transcriptional dynamics and estimate the joint probabilistic distribution of unspliced and spliced counts at steady state. Kinetic parameters in the CMEs are optimized by minimizing the Kullback–Leibler (KL) divergence between the inferred and experimentally observed joint distributions (Fig. 2B). Finally, *TopicVelo* estimates process-specific kinetic rates and computes corresponding process-specific transition matrices. These individual matrices are then combined to form a unified transition matrix estimate for each cell.

The steady-state RNA velocity methods have demonstrated utility in specific biological contexts. For example, they have been applied to infer T cell differentiation under clonal hematopoiesis [49] and trace cytotoxic and exhausted CD8+ T cell states in tumors [50]. The probabilistic topic approach adopted by *TopicVelo* has uncovered distinct transcriptional programs within mixed cell populations [51]. However, the foundational assumptions of these models impose substantial limitations. In particular, assuming constant splicing rates across genes and relying on identifiable steady-state populations can lead to inaccurate inference in heterogeneous systems or dynamic processes with incomplete trajectories. Although models like TopicVelo partially relax these constraints by modeling multiple transcriptional programs independently, steady-state methods remain suboptimal for capturing gene-specific kinetics and complex dynamic behaviors.

### Trajectory methods

*scVelo* [10] introduced a likelihood-based dynamical model for inferring RNA velocity that addresses complete gene-specific transcriptional dynamics, without relying on steady-state assumptions. This method solves full transcriptional dynamics by constructing phase trajectories, visual manifestations of the transcriptional dynamics (Fig. 2C and Supplementary Material 1). The trajectories are governed by an analytical solution of ODEs and illustrate how genes’ unspliced and spliced mRNA levels evolve over time as a function of the kinetic parameters. To estimate these dynamics, the dynamical model utilizes an efficient expectation maximization (EM) framework. This framework jointly estimates gene-specific kinetic parameters along with gene-specific latent time and transcriptional states. In the expectation step, latent time is assigned to cells by minimizing the distance between observed cell states and their corresponding positions along the current trajectory estimate. Transcriptional states are assigned by associating a likelihood value with respective segments of the phase trajectory. In the maximization step, kinetic parameters are optimized to define a phase trajectory that maximizes the log-likelihood of all observed displacements between cells and their inferred transcriptional states (Fig. 2C).

*MultiVelo* [14] extends the RNA velocity framework by integrating chromatin accessibility in the EM framework. As previously illustrated in steady-state context, the regulation of transcription process via chromatin accessibility is explicitly modeled into the ODE system in *MultiVelo*. This incorporation allows the model to account for upstream regulatory influences. The distinct phases or states of chromatin activity that a cell moves through as its inferred time progresses are modeled analogously to the stepwise transcription states used in *scVelo*’s dynamical model. Note that the fitted trajectory is extended to a 3D phase portrait, where chromatin accessibility constitutes an additional dimension alongside unspliced and spliced mRNA levels.

*UniTVelo* [17] adopts an EM framework similar to that of *scVelo* and introduces a radial basis function (RBF) to model transcriptional dynamics and quantify RNA velocity in a top–down manner. Instead of modeling gene expression as discrete transcriptional states, *UniTVelo* employs a spliced-oriented design that defines spliced abundance as a smooth time-dependent function using RBFs. It then applies a linear dynamical system to compute the corresponding unspliced abundance, preserving a continuous relationship between unspliced and spliced RNA levels. Additionally, in the expectation step, *UniTVelo* also introduces a gene-shared latent time, which unifies gene-specific latent times by aligning them based on a common cell order. This helps to resolve inconsistencies in inferred directionality across different genes and ensures a more coherent temporal structure.

*Dynamo* [18] uses metabolic labeling to facilitate the study of transcriptional dynamics. It observes transcriptional dynamics in real time, enabling the estimation of RNA velocity and kinetic parameters on an absolute time scale. *Dynamo* fits trajectories using nonlinear regression based on experimentally measured cell time, rather than employing an EM framework that treats cell time as a latent variable for joint inference. By incorporating real-time developmental information, this method allows for more accurate extrapolation of cell states across time and improves the biological interpretability of inferred dynamics. As a result, RNA velocity estimates and kinetic parameters are expressed in absolute temporal units, providing a more faithful reconstruction of dynamic transcriptional processes across cells.

As an alternative to EM-based approaches, *veloVI* [19] implements a variational autoencoder (VAE) framework for RNA velocity estimation. A VAE is a generative model that leverages the principles of Bayesian inference to learn the distribution of input data and generate new samples. In this context, *veloVI* first encodes the unspliced and spliced abundances into a latent cell representation. This latent representation, in turn, is used to encode gene-specific latent transcriptional state assignment and latent time for each cell. A transcriptional ODE, with a similar formulation of *scVelo*, is then utilized to reconstruct the input unspliced and spliced matrices by adopting learnt latent variables. The reconstruction process can be interpreted as mapping observed cell states onto a cell-shared trajectory, with latent time anchoring each cell’s position along that trajectory. This mechanism conceptually aligns with the notion of the maximization step in EM methods (Fig. 2C). By leveraging a variational inference framework, *veloVI* enables RNA velocity to be modeled as a posterior predictive distribution, thus allowing for explicit quantification of uncertainty in both latent variables and velocity estimates.

*VeloVAE* [20] applies a similar VAE framework, whereas it models cell-specific transcription rates through lineage-dependent ODEs. This model first encodes a gene-shared latent time and latent cell state for each cell. A neural network is then employed to infer adjustments to transcription rates based on each cell’s latent state. This method leverages the biological intuition that cells near each other in the latent space are likely to exhibit similar transcriptional kinetics [52]. Consequently, mRNA abundances are reconstructed based on the transcriptional dynamic ODEs in a similar manner to *veloVI*. However, since transcription rates are cell-specific, each cell effectively follows its own dynamic trajectory in phase space (Fig. 2D). In addition, *VeloVAE* can be further extended to include a branching ODE system, allowing the model to infer lineage bifurcations and learn cell type–specific kinetic parameters across distinct developmental paths.

*LatentVelo* [21] computes a low-dimensional representation of gene dynamics by embedding cells into a latent space within a VAE framework and directly estimates the trajectory in a low-dimensional phase portrait. Latent dynamics are learned through a structured neural ODE system, which captures the interactions among transcriptional components without explicitly modeling transcription, splicing, and degradation rates. An external latent regulatory state is also included, enabling the estimation of lineage development and lineage-specific kinetic parameters. This model jointly reconstructs low-dimensional embeddings and original high-dimensional expression profiles, allowing it to fit complex, nonlinear trajectories. Additionally, batch correction, temporal information, and chromatin accessibility can be incorporated into the latent dynamics learning process to further refine latent dynamics learning.

*Pyro-Velocity* [22] recasts RNA velocity estimation as a latent variable posterior inference task, leveraging automatic differentiation variational inference to perform fully Bayesian inference. The model is conditioned directly on raw count data. It estimates the posterior distributions of kinetic parameters and latent time, allowing for explicit quantification of uncertainty in both RNA velocity and kinetic estimates. Model optimization is in a similar manner to VAE-based methods, learning kinetic parameters and latent variables to reconstruct the input data. *cell2fate* [23] builds upon this Bayesian framework by introducing a modular design that decomposes transcriptional dynamics into multiple regulatory modules. Each of these modules is defined by its own activation timing and gene loadings. It models time-dependent transcription rates as a linear combination of these modules and accounts for additional complexities such as ambient RNA, overdispersion, and batch effects. Moreover, cell2fate extends applicability to spatial transcriptomics by mapping inferred regulatory modules to spatial tissue contexts.

As the most widely adopted RNA velocity methods, trajectory-based approaches have demonstrated substantial utility in resolving cellular differentiation trajectories, refining lineage inference, and integrating multi-omics data. For example, *scVelo* has been applied to elucidate oligodendrocyte precursor cell development in the human forebrain [53], capture unidirectional transitions in chronic lymphocytic leukemia [54], and reveal interactions between chromatin accessibility and transcriptional regulation during retinal differentiation [55]. Collectively, the trajectory-based approaches significantly extend the capabilities of RNA velocity beyond the limitations of steady-state methods and offer flexible modeling of complex, nonlinear transcriptional dynamics. Furthermore, these methods often incorporate latent time or regulatory states, which enhances their biological interpretability. However, these methods remain sensitive to incomplete or partial trajectories and rely on ODE formulations that may not fully capture multifaceted or branching kinetic processes.

### State extrapolation methods

*cellDancer* [24] infers RNA velocity for each cell by leveraging expression states from its neighboring cells and propagates a series of local velocities to provide single-cell resolution inference of transcriptional kinetics. For each gene, this model trains an independent deep neural network to calculate cell-specific kinetic rates. Future expression states are extrapolated by modeling short-term changes in both unspliced and spliced mRNA abundances, governed by transcriptional dynamics ODEs. The optimization objective is to maximize the global cosine similarity between extrapolated cell states and their observed neighbors in the phase portrait (Fig. 2E). The expected future state is selected as the neighbor with the highest cosine similarity, which serves as a guide for velocity vector refinement. *cellDancer* directly incorporates the local velocity vector into its training process and learns cell-specific reaction rates. This design effectively captures multi-rate kinetic regimes, thereby ensuring a more accurate and nuanced representation of transcriptional kinetics.

*DeepVelo* [25] employs a graph-based deep learning framework to estimate local velocity and extrapolate cell states within the high-dimensional splicing space. The method begins by identifying each cell’s KNN and encoding the resulting local neighborhood into a latent representation using a GCN. The GCN effectively captures local cell–cell relationships based on gene expression profiles. A downstream decoder network then predicts gene- and cell-specific kinetic parameters, which are used to extrapolate future cell states. In *DeepVelo*, transcriptional dynamics are learned by minimizing the cumulative displacement between predicted cell states and their expected neighbors (Fig. 2F). Unlike *cellDancer*, which relies solely on the most similar neighboring cells, *DeepVelo* incorporates both downstream and upstream neighbors to supervise the optimization of forward and backward velocity vectors for each cell. This approach thereby allows a more comprehensive inference of transcriptional dynamics.

*SymVelo* [26] is a dual-path framework that integrates high- and low-dimensional information through mutual learning to enhance RNA velocity estimation. The high-dimensional branch uses a neural ODE module to learn gene-specific kinetics in a manner similar to *DeepVelo*. In parallel, the low-dimensional branch adopts a *VeloAE*-inspired framework, learning RNA velocity in latent space. The inferred low-dimensional velocities are then used to supervise neighbor selection in the extrapolation branch. The two branches in *SymVelo* are aligned by first computing independent Markov transition matrices from each branch and then minimizing the divergence between them. This alignment strategy allows the model to combine the robustness of low-dimensional representation with the biological interpretability of high-dimensional dynamics. Such mutual learning strategy improves coverage across latent dimensions and enables intergene information sharing during representation learning.

Compared to other RNA velocity approaches, state extrapolation methods offer superior capacities to capture lineage heterogeneity by dynamically predicting future cell states beyond static transcriptional snapshots. Their ability to estimate local velocity vectors at single-cell resolution and extrapolate expression states allows for a more refined reconstruction of transcriptional kinetics across diverse cellular trajectories. A key advantage of this approach is its robustness in handling multi-rate kinetic regimes, where transcription, splicing, and degradation rates vary across cell subpopulations. For example, *cellDancer* has demonstrated strong performance in resolving transcriptional boost genes, such as *Hba-x* and *Smim1*, during erythroid maturation in mouse gastrulation [24]. These genes undergo sudden transcriptional upregulation in the middle of erythroid differentiation. This behavior poses a challenge for traditional RNA velocity models like *scVelo*, which often fail to capture the induction phases of such multiple-rate kinetic (MURK) genes, whereas *cellDancer* accurately inferred their dynamics.

Given these advantages, state extrapolation methods may hold particular promise for future applications in highly heterogeneous biological systems, such as tumor evolution, immune cell differentiation, and dynamic transcriptional regulation in response to environmental stimuli. By enabling single-cell resolution estimation of RNA velocity in complex multi-lineage contexts, these models have the potential to improve the precision of cell fate predictions in developmental biology and disease modeling. Nevertheless, the performance of state extrapolation methods is sensitive to the accuracy of neighbor selection, and the extrapolation process can be computationally demanding.

## Application of RNA velocity under various biological scenarios

Through its unique capability of predicting future cell states by analyzing unspliced and spliced mRNA ratios, RNA velocity has provided unprecedented insights into cellular dynamics across diverse biological systems. These applications predominantly fall into three scenarios: differentiation and development, diseased and injured microenvironments, and tumor microenvironments. Specifically, in developmental biology, RNA velocity has significantly advanced our understanding of complex lineage relationships and temporal hierarchies, spanning from early embryonic development to tissue-specific differentiation. In disease research, this technique has uncovered abnormal cellular transitions and disrupted developmental trajectories, offering insights into disease progression, impaired regeneration, and key regulatory pathways that may serve as therapeutic targets. In tumor research, RNA velocity has helped reveal intratumoral heterogeneity, plasticity in cancer cell states, and dynamic interactions between tumor cells and immune populations within the microenvironment. A comprehensive summary of RNA velocity applications across these biological contexts is presented in Table 3, highlighting representative cases and their major findings. In the following sections, we further detail the specific roles of RNA velocity in each scenario, analyzing its contributions to developmental biology, disease research, and tumor microenvironments.

**Table 3:** Summarization of RNA velocity applications across biological scenarios.

Categories	Tissue/Cells	Biological implications	Algorithms	Key findings	Ref.
Differentiation and development	Mouse embryonic cells	Asynchronous emergence; multiple subpopulations	*scVelo* (deterministic)	Identified three neural crest cell subpopulations and validated cellular trajectories from E3.5 to E13.5.	[56]
	Human forebrain	Rare population identification; temporal sequence; multiple developmental states	*scVelo* (dynamical)	Revealed temporal sequence of OPC specification and identified two distinct EGFR^+^ populations as OPC sources.	[53]
	Zebrafish neural stem cells	scRNA-Seq and scSLAM-Seq dual validation; alternative pathways; spatiotemporal diversity	*scVelo* (stochastic) *Dynamo*	Validated direct and proliferative differentiation trajectories.	[57]
	Zebrafish enteric nervous system	Neuronal subtype specification; spatiotemporal emergence patterns	*scVelo* (dynamical); *UniTVelo*	Identified distinct neuron subtypes with spatiotemporal emergence patterns.	[58]
	Human retina	Bidirectional development; multi-omic integration; temporal progression	*scVelo* (dynamical) *MultiVelo* (dynamical)	Revealed bidirectional developmental trajectories of retinal progenitor cells.	[55]
	Mouse medullary thymic epithelial cells	Branching development; precursor validation; experimental verification	*scVelo* (stochastic & dynamical)	Identified TAC-TECs as precursors to major mTEC subpopulations and predicted branching developmental model.	[59]
	Human bone marrow stromal cells	Hierarchical organization; multiple directions; driver gene identification	*scVelo* (stochastic)	Revealed two main developmental directions from MSSCs and identified key driver genes.	[60]
	Human Bone marrow	Lineage commitment; transitional progenitor states; NK cell ontogeny	*scVelo* (no model specified); *TopicVelo*	Identified transitional NK progenitors bridging HPCs and NK cells.	[51]
	Human B cells from multiple organs	Peripheral origin of thymic B cells; spatiotemporal B cell subset relationships	*UniTVelo*	Peripheral origin of thymic B cells confirmed by trajectory.	[61]
	Human subcutaneous adipose tissue	Active transitions; multiple sample types; scRNA-Seq and snRNA-seq analysis	*scVelo* (dynamical)	Validated adipocyte differentiation trajectories in WAT and SVF samples.	[62]
	Human small intestine	Sequential differentiation; unidirectional progression; stepwise maturation	*scVelo* (dynamical)	Mapped unidirectional cellular trajectory from stem cells to mature absorptive enterocytes.	[63]
	Human intestinal cells	Lineage differentiation; secretory cell specification; intracryptal maturation	*CellDancer*	Traced origin of differentiated intestinal cell types from intestinal stem cells.	[64]
	Human endometrial tissue	Epithelial-to-stromal transition	*scVelo* (dynamical), *CellDancer*	Revealed disrupted epithelial-mesenchymal transition in preeclampsia patients.	[65]
	Human endometrial tissue	Luminal-to-glandular differentiation; endometrial regeneration	*UniTVelo*	Luminal cells show high differentiation potential toward glandular cells.	[66]
	Human fetal lung tissue	Progenitor differentiation	*LatentVelo*	Uncover unexpected cell lineage transitions in developing epithelium.	[67]
	Mouse testis (spermatocytes)	Meiotic progression; transcriptional dynamics in spermatogenesis	*scVelo* (dynamical), *UniTVelo*	Revealed stage-specific regulators guiding pachytene progression in spermatogenesis.	[68]
	Mouse visual cortex neurons	Stimulus-dependent transcriptional dynamics; vision-dependent circuit refinement during development	*UniTVelo*	Immediate early gene versus late response gene waves identified.	[69]
	Tomato callus	Sequential states; developmental relationships	*scVelo* (dynamical); *Dynamo*	Elucidated developmental trajectory among three shoot primordia subtypes.	[63]
Diseases and injured microenvironments	Human & mouse monocytes	Early fate decision; alternative pathways; distinct lineages	*scVelo* (stochastic)	Revealed early bifurcation of monocytes into mo-DC and mo-Mac lineages.	[70]
	Human peripheral blood mononuclear cells	Population heterogeneity; SLE patient variation; disease correlation	*scVelo* (no model specified)	Examined transcriptional heterogeneity in SLE patients.	[71]
	Human placentas	State transitions; disease-specific populations; developmental divergence	*scVelo* (dynamical)	Showed developmental stalling of preeclamptic trophoblasts.	[72]

*(continued)*

**Table 3:** Continued.

Categories	Tissue/Cells	Biological implications	Algorithms	Key findings	Ref.
	Mouse and human lung alveolar epithelium	Treatment response; state redirection; therapeutic mechanism	*scVelo* (dynamical)	Revealed how HIF2 inhibition alters cell fate decisions.	[73]
	Human postmortem prefrontal cortex	Synaptic dysregulation; cell cycle acceleration; neurodegenerative trajectory	*scVelo* (stochastic); *veloVI*	Revealed accelerated synaptic and developmental disruptions in Alzheimer’s cortex.	[74]
	Mouse cardiac cells	Sequential transitions; Multiple population dynamics	*scVelo* (stochastic & dynamical)	Mapped transitions in fibroblast and macrophage populations postmyocardial infarction.	[75]
	Human diabetic foot ulcer keratinocytes	Healing dynamics; state fluidity; comparative analysis	*scVelo* (dynamical)	Revealed differences in cellular dynamics between healing and nonhealing DFUs.	[76]
	Human lung epithelial cells	Bidirectional trajectory; infection response; temporal stages	*scVelo* (no model specified)	Identified bidirectional differentiation between IS cells and BC during infection.	[77]
	Murine skin	Driver gene identification; Phase transitions; developmental potential	*scVelo* (dynamical)	Identified Lef1 as a critical driver gene in papillary fibroblast development.	[78]
Tumor microenvironments	Mouse T cells	Temporal hierarchy; stage-specific effects; T cell fate transition	*scVelo* (stochastic)	Revealed temporal hierarchy of epigenetic regulation during T cell differentiation.	[49]
	Human nonsmall cell lung cancer cells	Dual origins; treatment response; immune dynamics	*scVelo* (stochastic)	Validated two distinct origins of cytotoxic T cells in the tumor microenvironment.	[50]
	Mouse tumor-infiltrating lymphocytes	T cell exhaustion trajectory; spatial trajectory; origin identification	*scVelo* (stochastic)	Demonstrated developmental trajectory from lymph nodes to tumors.	[79]
	Human nonsmall cell lung cancer cells (NSCLCs)	State evolution; multiple transitions; microenvironment influence	*scVelo* (dynamical)	Uncovered evolution pattern of neutrophil subtypes in NSCLC.	[80]
	Human prostate cancer epithelial cells	Tumor heterogeneity; directional transformation; origin resolution	*scVelo* (dynamical)	Demonstrated NEPC cells originate exclusively from luminal-like malignant cells.	[81]
	Human colorectal polyp cells	Contrasting trajectories; precancerous dynamics	*scVelo* (no model specified)	Validated distinct cellular origins of different polyp types.	[82]
	Human chronic lymphocytic leukemia peripheral blood mononuclear cells	Unidirectional progression; state transition; irreversible fate	*scVelo* (no model specified)	Revealed unidirectional fate progression in the lymph node microenvironment.	[54]
	Human and mouse pancreatic ductal adenocarcinoma cells	PI3Kδ inhibition mechanism; development disruption; side effect explanation	*scVelo* (no model specified)	Revealed how PI3Kδ inhibition affects *T*_reg_ development.	[83]
	Human primary central nervous system lymphoma cells	Clonal heterogeneity; developmental diversity; subclone evolution	*scVelo* (dynamical)	Identified developmental paths between malignant B cell clusters.	[84]
	Human glioma stem cells	Hypoxia-induced cell state transitions; epigenomic and transcriptomic remodeling	*MultiVelo*	Revealed hypoxia-induced reversal of cell state trajectories in multiple clusters, identified regulatory mechanisms through gene models of activation/repression.	[85]
	Human myeloid and T lymphocytes	Immune cell fate determination	*Dynamo*	Revealed distinct lineage commitment pathways across immune archetypes.	[86]

### Differentiation and development

Understanding cellular differentiation and lineage specification is a fundamental objective of single-cell omics research, as it provides crucial insights into how cells acquire distinct identities and functions across developmental stages. For instance, in the context of neural embryonic development, RNA velocity has enabled identification of distinct neural crest subpopulations and precise reconstruction of developmental trajectories, surpassing heuristic approaches by capturing subtle cell-state transitions [56, 58]. It has also elucidated directional lineage progression and regulatory gene dynamics during human forebrain oligodendrocyte precursor cell specification [53]. Mitic *et al*. utilized RNA velocity to uncover dynamic transitions of neural stem cells in the adult zebrafish telencephalon under both homeostatic and regenerative conditions [57]. Additionally, a study of human retinal development employed *MultiVelo*, integrating RNA velocity with chromatin accessibility data to refine analyses of retinal progenitor differentiation trajectories [55]. Stromal and immune cell studies leveraged RNA velocity to trace the differentiation trajectory of medullary thymic epithelial cells [59]. Similarly, Li *et al*. applied RNA velocity to dissect lineage relationships among human bone marrow stromal cells, identifying key regulatory genes along their differentiation paths [60]. *TopicVelo* was employed to reconstruct the differentiation trajectory from bone marrow precursors to classical Natural Killer (NK) NK cells, successfully capturing the lineage commitment that *scVelo* failed to resolve [51]. In studying B cells from multiple organs, *UniTVelo* confirmed the peripheral origin of thymic B cells [61]. In tissue-specific differentiation, RNA velocity has enabled identification of distinct adipocyte subpopulations and tracking of adipocyte differentiation trajectories [62]. It has also reconstructed absorptive enterocyte differentiation in the human intestine [63]. Additionally, *CellDancer* was used to trace the origin of differentiated intestinal cell types from intestinal stem cells [64]. RNA velocity has also been applied to human endometrial tissue studies. In pathological conditions, it revealed disrupted epithelial–mesenchymal transition in preeclampsia patients [65]. In normal regeneration, it showed that luminal cells exhibit high differentiation potential toward glandular cells [66]. *UniTVelo* has improved trajectory inference during pachynema progression in mouse testis spermatocytes and identified two waves of transcription in mouse visual cortex neurons [68, 69]. *LatentVelo* was used to uncover unexpected cell lineage transitions in human fetal lung development [67]. Extending beyond animal systems, its application in plant development delineated shoot primordia differentiation in tomato callus [87], underscoring its versatility across biological domains. Collectively, RNA velocity has provided quantitative insights into cellular transitions, emphasizing its critical role in deciphering differentiation and developmental processes.

### Diseased and injured microenvironments

Building upon insights from normal development, RNA velocity has been extensively applied to pathological conditions, providing key insights into immune system disorders, developmental disorders, tissue repair, and regeneration. In immune disorders, this technique characterized monocyte fate decisions during inflammation [70] and revealed altered developmental trajectories of monocyte and T cell subsets in systemic lupus erythematosus [71]. In developmental disorders, RNA velocity demonstrated developmental stalling and abnormal endothelial cell differentiation in preeclampsia [72] and traced dynamic cell fate trajectories during lung epithelium regeneration [73]. Integration with tools such as *veloVI* refined predictions of neuronal state transitions and identified synaptic dysfunction associated with Alzheimer’s disease [74]. Extending these insights into regenerative contexts, RNA velocity dissected tissue repair mechanisms and pathological remodeling events. It revealed bidirectional plasticity between fibroblast and macrophage populations in cardiac fibrotic microenvironments [75]. RNA velocity also inferred distinct differentiation patterns between healing and nonhealing diabetic foot ulcers [76]. Furthermore, it mapped macrophage differentiation trajectories following myocardial infarction, predicting terminal states and monocyte origins​ [77]. It also supported continuous models of fibroblast activation during wound healing [78]. In summary, these studies highlight RNA velocity’s ability to capture aberrant cellular transitions and disrupted developmental trajectories in disease contexts, offering mechanistic insights that enhance our understanding of pathophysiology and inform therapeutic strategies.

### Tumor microenvironments

As one of the most complex and recalcitrant frontiers in disease research, tumors present unique complexities due to their dynamically evolving microenvironments—heterogeneous ecosystems in which cancer cells, immune populations, and stromal components interact intricately. RNA velocity has emerged as a crucial tool for dissecting these interactions, offering insights into immune cell dynamics, cancer cell plasticity, and therapeutic responses within tumor microenvironments. Regarding immune cell dynamics, RNA velocity has provided multiple insights. It elucidated epigenetic regulation during T cell differentiation [49] and characterized distinct differentiation trajectories of CD8+ T cells [50]. Additionally, it identified a stem-like T cell reservoir within lymph nodes that sustains antitumor immunity [79] and mapped neutrophil differentiation in nonsmall cell lung cancer [80]. In studies of cancer cell plasticity, RNA velocity has clarified cellular origins of neuroendocrine prostate cancer [81]. It also demonstrated distinct developmental pathways in colorectal polyps [82] and revealed directional progression in chronic lymphocytic leukemia within lymphoid tissues [54]. Furthermore, RNA velocity provided mechanistic insights into therapeutic responses, demonstrating how PI3Kδ inhibition disrupts regulatory T cell development while promoting inflammatory T cell subsets, informing potential dosing strategies [83]. Additional applications in tumor contexts included analysis of clonal heterogeneity and developmental diversity in primary central nervous system lymphoma [84] and identification of hypoxia-induced cell state transitions in glioma stem cells [85]. Furthermore, *Dynamo* was used to reveal distinct lineage commitment pathways across immune archetypes in a study of bone metastasis ecosystems [86]. Despite its utility, challenges remain, including the frequent absence of ancestral cells in tumor samples and mutation-induced aberrant splicing, underscoring the necessity of complementary approaches such as chromosomal aberration analysis for more robust trajectory inference [84]. Despite these limitations, RNA velocity remains instrumental in dissecting tumor evolution and immune interactions, opening avenues for novel therapeutic strategies.

In conclusion, RNA velocity has demonstrated remarkable versatility and power across developmental biology, disease research, and cancer studies. Across all these fields, it has been particularly valuable for resolving cellular hierarchies, validating developmental trajectories, and identifying key molecular drivers of cell fate decisions. As technological advances continue to address current limitations, RNA velocity analysis is poised to remain a key tool in understanding cellular dynamics in both normal and pathological conditions, ultimately contributing to advances in regenerative medicine, disease treatment, and cancer therapy. As our application summary (Table 3) illustrates, a growing number of RNA velocity methods are demonstrating their value in specific biological applications. Tools such as *UniTVelo*, *Dynamo*, and *cellDancer* have already been successfully applied in diverse research contexts, showcasing their unique strengths. Nonetheless, *scVelo* currently remains the most prevalent tool in practice, largely due to its early availability and demonstrated versatility in a vast number of trajectory inference and differentiation prediction studies. However, several studies have highlighted critical limitations in both steady-state (deterministic and stochastic) and trajectory (dynamical) models of *scVelo*, demonstrating their shortcomings in accurately resolving developmental dynamics and predicting future cell states in complex biological systems [11–13, 51, 88].

## Discussion

The advent of RNA velocity has revolutionized single-cell transcriptomics by enabling dynamic predictions of cellular states through the temporal interplay of spliced and unspliced mRNAs. From its foundational models (*Velocyto* and *scVelo*) to advanced frameworks integrating multi-omics data or deep learning, these methods have illuminated cellular trajectories in developmental biology, disease progression, and tumor ecosystems. By capturing transcriptional kinetics, RNA velocity has resolved lineage hierarchies, identified fate-determining genes, and even challenged traditional paradigms—such as the unidirectional evolution of leukemia cells in lymphoid tissues. Its applications span diverse biological scales, from embryonic patterning to immune cell exhaustion, solidifying its role as a cornerstone of dynamic cellular analysis.

Recent developments have expanded splicing-kinetic-based RNA velocity frameworks to broader biological contexts and more accurate dynamic inference. *Protaccel* [89] incorporates protein-level dynamics into steady-state models, enabling prediction of protein production rates from transcriptomic data. SIRV [90] integrates spatial transcriptomics to reconstruct tissue-specific dynamics through spatially aligned velocity inference. *DeepKINET* [91] applies deep learning to estimate splicing and degradation rates at single-cell resolution, offering insights into post-transcriptional regulation. Cell cycle–specific models such as *DeepCycle* [92] and *VeloCycle* [93] infer RNA velocity in a periodic framework, revealing continuous phase progression and variations in cell-cycle speed. In addition, several methods extend RNA velocity modeling beyond splicing kinetics altogether. For example, a generalized model of *Dynamo* [18] uses metabolic labeling to estimate total RNA velocity without modeling splicing explicitly. *TFvelo* [94] infers gene-specific dynamics from transcription factor–target relationships, while *scKINETICS* [95] models phenotype transitions through regulatory network–driven differential equations. Collectively, these advancements extend RNA velocity beyond traditional assumptions, opening new opportunities for studying transcriptional regulation, temporal coordination, and multi-omic integration in dynamic systems.

Despite these transformative insights, critical challenges persist. The reliance on steady-state assumptions, technical noise in single-cell data, and limitations in visualizing high-dimensional dynamics often constrain the accuracy and generalizability of RNA velocity predictions, particularly in systems with transient transcriptional states or heterogeneous kinetics. Conventional workflows may inadvertently obscure biological signals through preprocessing steps, while projections onto low-dimensional embeddings risk oversimplifying complex trajectories. These limitations highlight the need for methodological refinement and integrative validation.

### Challenges of complex transcriptional dynamics in RNA velocity estimates

Several recent reviews [11–13, 33, 96] have comprehensively discussed critical challenges that lead to failures in capturing transcriptional dynamics through RNA velocity methods, particularly regarding two conventional tools: *Velocyto* and *scVelo*. These studies highlight that many models rely on simplifying assumptions of transcriptional dynamics, such as steady-state kinetics or constant kinetic rates. However, these assumptions often do not hold in biological systems with complex transcriptional kinetics, resulting in incorrect inference of full transcriptional dynamics [13]. These inherent limitations have been a primary driver for the continuous innovation and development of new RNA velocity methods designed to address more complex biological scenarios.

A core challenge in RNA velocity analysis stems from the inherent complexity of biological systems. Many genes exhibit multiple kinetic regimes, such as transcriptional boosts within specific cell subpopulations [88, 97] or lineage-dependent kinetics patterns [8, 10]. This manifests as genes that display multiple trajectories and a secondary boost of induction in phase space. Traditional steady-state methods, which infer kinetics that are only present in steady-state populations by using linear regression, struggle to resolve dynamics in heterogeneous subpopulations that deviate from steady state. Methods such as *TopicVelo* partially relax this assumption by identifying distinct biological processes and modeling dynamics separately for each process.

Trajectory methods assign cell time and concurrently fit phase trajectories to solve the full dynamics of transcription. *scVelo* (dynamical model), *veloVI*, and *Pyro-Velocity*, formulate transcriptional ODEs in a step-wise manner, where cells are assigned to transient states of induction and repression and two steady states (active and inactive). The induction and repression stages of the phase trajectory are fitted using nonsteady-state cells, thereby partially reducing the interference from heterogeneous steady-state cells, which are often composed of highly differentiated mature subpopulations. However, stepwise trajectory methods suffer from limitations in capturing the complex kinetics within induction and repression stages. To more precisely capture such complexities, subsequent trajectory methods have incorporated several advancements. The dynamical model of *MultiVelo* extends transcriptional ODEs by incorporating chromatin accessibility, enabling it to model time-varying transcriptional rates. *VeloAVE* and an extension of *veloVI* estimate lineage-dependent or time-dependent transcription rates directly from the expression profile. *VeloVAE* further enables the construction of cell-type transition graphs and fits branching ODEs tailored to bifurcating cell populations. *LatentVelo* reformulates transcriptional dynamics into structured neural ODEs and captures latent dynamics in a latent embedding of cell states. In addition, by incorporating state-dependent transcriptional regulation, *LatentVelo* effectively estimates complex lineage- and time-dependent kinetic rates. *cell2fate* employs a modular approach, disentangling cellular dynamics into multiple modules and modeling time-dependent transcription rates as a linear combination of these modules.

State extrapolation methods integrate inferred RNA velocity into dynamic learning by inferring cell-specific kinetics from neighboring cells rather than fitting global dynamics. Specifically, *cellDancer* infers cell-specific kinetics by estimating the unspliced and spliced velocities in phase space, whereas *DeepVelo* and *SymVelo* estimate velocities within the high-dimensional gene expression space, such as the high-dimensional spliced space. By accurately identifying the expected future state of each cell as a supervisory signal, these methods are able to capture subtle kinetic variations within heterogeneous populations and across multiple lineages.

Another significant challenge arises from insufficient observations of transcriptional dynamics within specific subpopulations, posing challenges for the accurate estimation of RNA velocity for certain genes [13]. This occurs when a gene is active only during a brief window of the observed process, such as monotonous upregulation at the end or downregulation at the beginning of a developmental process. For steady-state methods, the lack of observations in steady-state populations violates the basic steady-state assumptions, leading to inaccuracies in linear regression. Moreover, incomplete dynamics often result in cells in phase space being distributed along a straight line rather than a curve. Trajectory methods, such as the dynamical model of *scVelo*, therefore struggle to determine whether a trajectory is in the upregulation or downregulation phase. To overcome this, *MultiVelo* incorporates chromatin accessibility to help infer transcriptional states. Methods like *UniTVelo* assign a unified cell time and aggregate dynamic information across all genes, enhancing the identification of transcriptional states for genes with incomplete dynamics. The use of unified cell time also helps mitigate overfitting from high technical noise and complex gene activities. *Dynamo* takes this further by directly extracting cellular real-time information from metabolic labeling data. State extrapolation methods, due to their local modeling nature, do not rely on observing the complete dynamic curve. Therefore, they are often more robust when handling partially observed dynamics.

### Challenges in data preprocessing

The standard RNA velocity workflow begins by distinguishing unspliced and spliced matrices from raw data. However, this binary classification of transcripts overlooks potential transient and terminal isoforms of RNAs arising from alternative splicing mechanisms [11]. Such informative ambient RNAs can be detected by quantification tools, yet they are excluded from most methods. *cell2fate* is one of the few that attempts to account for ambient RNAs. Furthermore, the quantified count matrices inherently contain substantial noise arising from the low-copy number regime in single-cell RNA synthesis, as well as technical noise intrinsic to scRNA-seq measurements [11, 13, 98]. Conventional RNA velocity methods address these issues by employing cell size normalization and KNN imputation to remove noise, alongside filtering for high-quality cells and highly variable genes to facilitate kinetics prediction and velocity inference. Methods such as *VeloAE*, *LatentVelo*, and *SymVelo* further attempt to denoise data by encoding unspliced and spliced counts into a low-dimensional space to infer latent dynamics. However, it’s important to consider that some “noise” in single-cell data might be informative, reflecting the discrete stochastic nature of gene expression [99, 100]. Traditional preprocessing steps like count normalization can diminish the interpretability of these discrete data [11, 101, 102], and KNN imputation has been shown to potentially introduce distortions in both RNA velocity estimates and visualizations [11, 13]. Recognizing these considerations, methods such as *Pyro-Velocity*, *cell2fate*, and *TopicVelo* directly leverage unprocessed discrete raw counts for dynamics inference. These tools are often designed with built-in mechanisms to model the discrete nature, stochasticity, and noise inherent in such raw data. By doing so, these approaches aim to avoid the aforementioned pitfalls, diminish the reliance on *ad hoc* parameter tuning in data preprocessing, and model the inherent stochasticity of gene expression more faithfully. Currently, a comprehensive understanding of the optimal data input strategy and its varying impacts is limited. Thus, systematic and comprehensive benchmarking is crucial to establish when to use each approach and to clarify its effects across diverse noise levels and biological contexts.

### Challenges and critical interpretation of RNA velocity visualization

Projecting RNA velocity vectors onto low-dimensional embeddings for visualization is a standard practice in the field. However, this approach can obscure meaningful dynamics or introduce artifacts that do not accurately reflect the underlying biology [11, 13]. Both local neighborhoods and the global topology heavily depend on the construction of the KNN graph, which is susceptible to noise and lacks interpretability in terms of cellular relationships. Therefore, when exploring cellular developmental trajectories or potential dynamics, it is strongly recommended not to solely rely on streamline plots in 2D embeddings. Instead, a multi-faceted approach to scrutinizing these visualizations is essential. This involves comparing velocity projections across various dimensionality reduction methods to assess the consistency of observed patterns and considering the use of embedding techniques specifically designed to better preserve single-cell data topology [103]. More importantly, visualizations should always be contextualized with established biological knowledge, such as known cell fates or marker gene dynamics. In addition, the interpretation can be strengthened by considering model-derived outputs and associated downstream analyses, such as latent variables (cell states or developmental time), driver gene analyses, and velocity uncertainty quantifications. For some methods that learn a dynamics-informed embedding, such as *LatentVelo*, the resulting visualizations may more faithfully represent cellular trajectories.

### Navigating challenges and toward best practices

The successful application of RNA velocity methods requires a careful navigation of the challenges discussed and an informed approach to method selection and interpretation. While no single tool is universally optimal, researchers can adopt several best practices to enhance the reliability and biological insight derived from their analyses.

Firstly, understanding the interplay between the biological system’s complexity and the chosen model’s assumptions is paramount. Prior biological knowledge often hints at such complexity, like expectations of rapid cell changes, strong cell cycle effects, or high cell plasticity. Concurrently, preliminary exploratory data analysis can offer direct visual clues. For instance, varied lineage marker expression might show mixed cellular processes. Similarly, unusual gene phase portrait shapes (like clusters or loops, rather than simple curves) can suggest complex kinetics, such as multi-rate kinetics or transcriptional bursts, which basic models may not capture.

For simpler, well-defined biological systems, established RNA velocity methods are often adequate with good interpretability and efficiency. This might include classical steady-state models like *Velocyto* or widely used *scVelo* models (steady-state or basic dynamical). However, systems exhibiting the aforementioned complex characteristics or those with incomplete dynamics pose challenges. In such complex or highly heterogeneous systems (e.g. tumor microenvironments and developmental bifurcations), basic models may be insufficient. Instead, consider advanced trajectory methods that can model variable or lineage-specific rates or state extrapolation methods that infer kinetics for each cell. However, without comprehensive, standardized benchmarking across diverse datasets and biological scenarios, it is difficult to pinpoint a single “best” method for all such complex situations. Examining published application examples can offer valuable insights into how different methods perform and are chosen for specific complex biological contexts. For instance, application studies (Table 3) show that *UniTVelo* is useful in diverse developmental and regenerative contexts, potentially due to its unified time inference. Furthermore, methods that incorporate additional omics data can provide enhanced insights. *MultiVelo* excels by integrating epigenomic context (e.g. in retinal development or glioma studies), while *Dynamo* offers distinct advantages when metabolic labeling data is available. *TopicVelo* also demonstrates strength in dissecting distinct biological processes within mixed cell populations.

Secondly, critically evaluating data quality is essential for robust RNA velocity analysis. Key indicators include Unique Molecular Identifier (UMI) counts per cell, overall data sparsity, and the clarity of unspliced/spliced read separation. Dealing with inherent scRNA-seq noise and sparsity requires a careful strategy. Traditional preprocessing aims to mitigate these issues but can also introduce biases or obscure true biological stochasticity. An alternative is to use models designed for raw or minimally processed counts (e.g. *Pyro-Velocity*, *cell2fate*, *TopicVelo*), which may better preserve biological variability, particularly for higher-quality datasets (e.g. high UMIs). For noisier or sparser data, another effective approach involves RNA velocity tools that learn dynamics in a denoised latent space or incorporate other noise-robust mechanisms within their framework (e.g. *VeloAE*, *LatentVelo*, *SymVelo*).

Thirdly, visualization via low-dimensional embeddings, while intuitive, requires cautious interpretation. Streamline plots should not be the sole basis for conclusions. It is crucial to support these visual explorations by examining model-derived outputs (inferred kinetics, latent variables like cell states or time), associated downstream analyses (driver gene identification, uncertainty quantification where available, such as in *veloVI*, *Pyro-Velocity*, or *cell2fate*), and by comparing projections across different dimensionality reduction techniques or considering embeddings better suited for preserving single-cell data topology. Crucially, interpretations should be contextualized with existing biological knowledge to ensure biological plausibility.

In summary, effective RNA velocity analysis demands thoughtful consideration of the research question, system and data specifics, tool capabilities (Tables 1 and 2), and a commitment to critical interpretation and validation. To further advance the field and guide optimal method selection, the development of comprehensive, standardized benchmarks is crucial. Such benchmarks should involve fairly comparing multiple methods across diverse biological scenarios, ideally using datasets with clear ground-truth cellular dynamics and employing a richer set of quantitative evaluation metrics beyond visual assessment. Until such community standards are established, careful in-house method comparison and staying updated with emerging best practices remain key for robustly harnessing RNA velocity for profound insights into the dynamic cellular world.

**Key Points**

- This review systematically categorizes RNA velocity computational tools into three paradigms: steady-state, trajectory-based, and state extrapolation methods, outlining their assumptions, modeling strategies, and kinetic inference approaches.
- The article provides a comprehensive comparison of representative RNA velocity models, highlighting their innovations, technical frameworks, and how they handle cell-specific transcriptional dynamics.
- Practical guidance is offered for the selection and application of RNA velocity methods, including discussions of preprocessing pitfalls, model limitations, and trajectory visualization artifacts.
- A detailed summary of biological applications is presented, demonstrating how RNA velocity has been used to study cellular differentiation, immune regulation, disease progression, and tumor microenvironmental dynamics.
- The review outlines future directions and current challenges, emphasizing the need for robust inference under heterogeneous kinetics, integrative multi-omics modeling, and broader validation in complex biological systems.

## Supplementary Material

Supplementary_Material_1_bbaf339

## Data Availability

Not applicable.
