# Supplementary material for: Paradigms, innovations, and biological applications of RNA velocity: a comprehensive review
Source: Brief Bioinform. 2025 Jul 16;26(4):bbaf339. doi: 10.1093/bib/bbaf339 (PMC12265890; doi:10.1093/bib/bbaf339)
Supplement: Supplementary_Material_1_bbaf339 [file supplementary_material_1_bbaf339.docx]

**Mathematical modeling of splicing-kinetic-based transcription dynamics**

Numerous computational methods have been developed for inferring RNA velocity, whereas most of them were built based on a similar ODE system in describing the splicing-kinetic-based transcription process. The transcriptional dynamics process encompasses the induction (‘on’ phase) and repression (‘off’ phase) of unspliced precursor mRNAs *u*(*t*) with rates *α*, their splicing into mature mRNAs *s*(*t*) at a rate *β* (which involves intron removal from pre-mRNAs and exon joining to produce spliced mRNAs), and eventual degradation at a rate *γ*.

$$\emptyset\overset{\alpha}{\to}u(t)\overset{\beta}{\to}s(t)\overset{\gamma}{\to}\emptyset$$

Here, we introduce a classic transcriptional ODE formulation from *scVelo*. In this system, splicing rates *β* and degradation rates *γ* are presumed to be constant (time independent) for each gene, and transcription rates *α*^(^*^k^*^)^ are assumed to be state-dependent constant for each gene. The gene-specific rate equations can be depleted with the ODE system:

$\begin{aligned} &\frac{du(t)}{dt}=\alpha^{(k)}-\beta u(t), \\ &\frac{ds(t)}{dt}=\beta u(t)-\gamma s(t), \end{aligned}$ (1)

which describing the evolution of mRNA abundances over time. The time derivative of mature spliced mRNA, termed RNA velocity, is commonly denoted as $v(t)=\frac{ds(t)}{dt}$. By treating transcription, splicing and degradation as probabilistic events, the transcriptional dynamics can be described with a Chemical Master Equation (CME), which gives full probability distribution over the counts of all reacting species:

$\begin{aligned} \frac{\partial p(u,s,t)}{\partial t}=&\alpha^{\left( k \right)}\left[ p\left( u-1,s,t \right)-p\left( u,s,t \right) \right], \\ &+\beta\left[ \left( u+1 \right)p\left( u+1,s-1,t \right)-up\left( u,s,t \right) \right], \\ &+\gamma\left[ \left( s+1 \right)p\left( u,s+1,t \right)-sp\left( u,s,t \right), \right]. \end{aligned}$ (2)

The analytical solution to (1) can be solved as following, to construct a phase portrait trajectory by specifying the values of *u*(*t*) and *s*(*t*) at different time points:

$\begin{aligned} &u(t)=u_{0}e^{-\beta\tau}+\frac{\alpha^{(k)}}{\beta}(1-e^{-\beta\tau}), \\ &s(t)=s_{0}e^{-\gamma\tau}+\frac{\alpha^{(k)}}{\gamma}(1-e^{-\gamma\tau})+\frac{\alpha^{(k)}-\beta u_{0}}{\gamma-\beta}(e^{-\gamma\tau}-e^{-\beta\tau}),\tau=t-t_{0}^{(k)}, \end{aligned}$ (3)

where *s*_0_ and *u*_0_ are the unspliced and spliced abundances at the phase switching time point $t_{0}^{(k)}$ in the transcriptional state *k*. *u*(*t*) and *s*(*t*) denote the normalized levels of unspliced and spliced mRNA, respectively, in a cell at a specific time *t*. Generally, the time point for each cell is challenging to determine in the absence of auxiliary experiment approaches (e.g. metabolic labeling), making *t* an unobserved latent variable. Similarly, the cellular transcriptional state *k* is also unobserved, and the kinetic parameters $\theta=(\alpha^{(k)},\beta,\gamma)$ are commonly not directly measured in experiments.

Using this dynamical system, *scVelo* employs an Expectation-Maximization (EM) framework to fit the parameters in Equation (3). Notably, *scVelo* defines the kinetic parameters as gene-specific, with the transcription rate modeled as stepwise. This formulation of the transcriptional ODEs directly reflects the concepts of uniform kinetics across all cells. In newly developed RNA velocity tools, the ODE system has been further restructured (e.g., by coupling transcription regulations in ODE system) or kinetic parameters have been customized (e.g., made cell- and gene-specific) to capture more complex biophysical processes.
